# Supplementary material for: Fung-AI: An AI/ML-driven pipeline for antifungal peptide discovery
Source: PLoS Comput Biol. 2026 Jun 15;22(6):e1014105. doi: 10.1371/journal.pcbi.1014105 (PMC13289945; doi:10.1371/journal.pcbi.1014105)
Supplement: S1 Text — (PDF) [file pcbi.1014105.s003.pdf]

Fung-AI: An AI/ML-driven pipeline for antifungal peptide discovery

S1 Text

Experiment

In this section, we present the experiment we designed to train and test the Fung-AI model.

Setup

All model building, training, and testing was implemented in Python version 3.9.13 using the libraries Tensorflow version 2.13.0 [1], Keras version 2.13.0 [2], and Keras-TCN version 3.5.0 [3] on one GeForce GTX 1080 Ti graphical processing units (GPUs) on a Linux operating system.

Classifier Training

Four classifiers were trained for this task. Each model was trained for 20 epochs with a batch size of 32. The Adam optimizer [4] was used for all models with the default learning rate and the binary cross entropy loss function (see equation 1).

$$\mathcal{L} = -\frac{1}{N}\sum_{i}^{N} y_i \log(\hat{y}_i) + (1 - y_i)\log(1 - \hat{y}_i) \qquad (1)$$

GAN Training

Autoencoder pretraining was done using the Adam optimizer [4] with a learning rate of 0.01 until the model reached a stable state. Training occurred for 100 epochs with a batch size of 256, followed by 50 epochs with a batch size of 64, where the weights of the first two layers were frozen to ensure a high degree of reconstruction. The autoencoder was trained using the sum of the sparse categorical cross entropy and the KL divergence (see equation 2) for the distribution of the encoder compared to a standard normal distribution, where μ and σ are the mean and standard deviation of the encoding vector [5].

$$\mathcal{L}_{KL} = \sum_{x \in X} P(x)\log\left(\frac{P(x)}{Q(x)}\right) = \log(\sigma) + \frac{1+\mu^2}{2\sigma^2} - \frac{1}{2} \qquad (2)$$

GAN training occurred in two stages based on number of discriminator training loops, for a total of 385 epochs. In both stages, we selected the Adam optimization algorithm. The learning rate for the generator was 1e-3 and for the discriminator was 2e-4 with $\beta_1 = 0.5$ and $\beta_2 = 0.9$ and a gradient penalty $\lambda = 10$. The learning rate for the discriminator was chosen to avoid mode collapse. All epochs were trained with a batch size of 128 and five generator epochs. The first 40 epochs had 5 discriminator epochs and the remaining 345 epochs used 3, as the discriminator was learning too quickly on each batch and needed to be slowed down.

While training the model, the 1D-BiLSTM antifungal peptide classifier trained using the BLOSUM embeddings was used to monitor the output of GAN and thereby also monitor training progress. While the model could have been trained for longer, it was stopped after 345 epochs as we were consistently seeing batches evaluated by the antifungal peptide classifier exceeding 50% predicted

antifungal. Therefore, on the final GAN epoch, we selected the last model to produce in excess of 50% predicted antifungal peptides.

Antifungal Peptide Classifier

Three antifungal peptide classifiers were selected from the literature to be implemented for the purpose of monitoring the training of the GAN and the process of down selecting the generated peptides. One model was based on temporal convolutional networks (TCNs) [8] using the architecture described in Singh et al. [6] (**Fig A**). The classifier began with an embedding layer applied to 3-mers of amino acids with a moving window of one and an embedding space of 200. The vocab size for this was 9265 (9261 for all the permutations of amino acids plus [START], [END], [UNK], and [EMPTY]). The embedding layer was followed by a 1D spatial dropout layer with a dropout rate of 0.25, which fed into two sequential TCN layers with filters of 128 and 64. Both had strides of (1, 2, 4), ReLU activations, dropouts of 0.25, and kernels of size 3. Following the TCN layers was a global average pooling layer, then finally two dense layers of size 16 and 1, with ReLU and sigmoid activation functions, respectively. A dropout layer of 0.25 separated the two fully connected dense layers. The model achieved an accuracy of 86.8%, AUC of 92.5%, and F1-score of 86.1% on our antifungal peptide datasets.

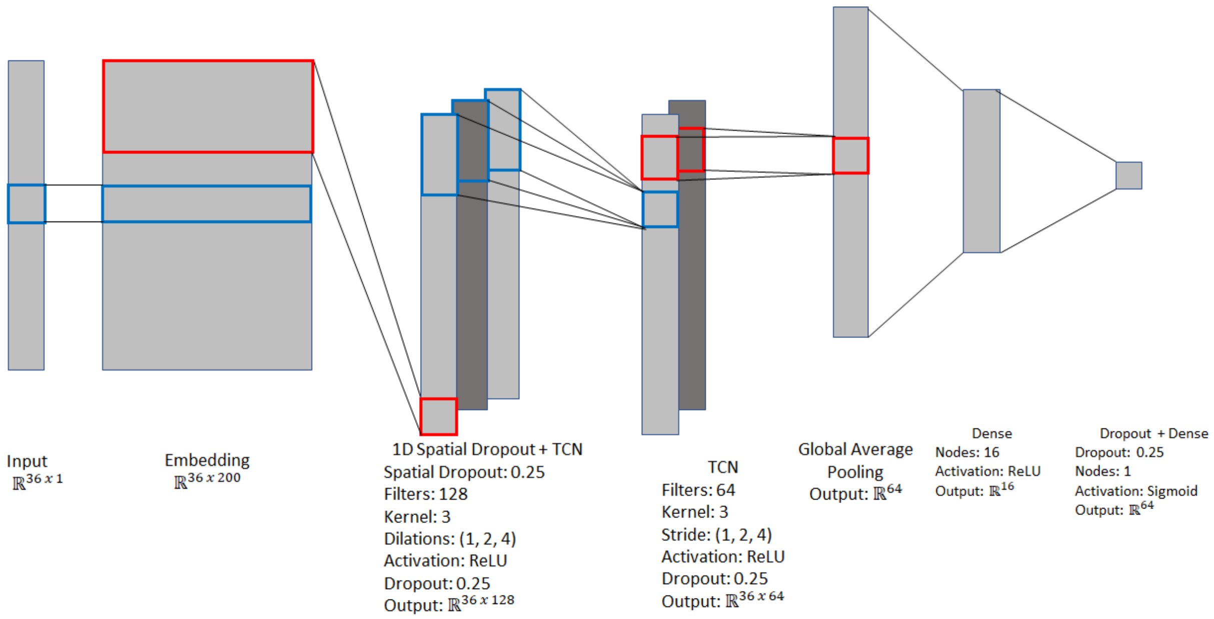

Fig A. Architecture of the first antifungal peptide classifier based on the one described in Singh et al. *[6]*, **using a temporal convolutional network (TCN).**

The other two models used the architecture described in Sharma et al. [7], which is based on a CNN-biLSTM architecture. Rather than using an input of 3-mers, the input was the sequence of amino acids. As a result, the vocab size was 25 (21 + 4 to account for [START], [END], [UNK], and [EMPTY]). The two variants were differentiated by the input and the presence of an embedding layer and the amino acid tokens were used as input. The first model took as input the one-hot encoded versions of the amino acids and with a vector dimension of 25, forgoing the need for an

embedding layer. The second model used an embedding layer with a dimension of 20 and was pre-seeded with the BLOSUM weights [9]. Both then fed into a convolution layer with a filter size of 128, kernel size of 8, stride of 1, and activation of ReLU. Next was an average pooling layer of size 2 and stride 2, followed by batch normalization and dropout of rate 0.25. The output fed into a bidirectional LSTM with 256 nodes and returned on the sequences, providing 256 dimensional vector for each token input. A global average pooling was applied across the sequence, followed by batch normalization, and dropout with a rate of 0.2. These steps were followed by two blocks of dense layers, batch normalization, and dropout. The first block had 256 nodes, a ReLU activation function, and a dropout of 0.15. The second block had 16 nodes, a ReLU activation function, and a dropout of 0.05. The final layer was a dense layer with a sigmoid activation function. The complete architecture is shown in **Fig B**. On our antifungal peptide dataset, the first model using the one-hot encodings achieved an accuracy of 85.5%, AUC of 92.1%, and F1-score of 84.6%. The second model with input based on the BLOSUM weights achieved an accuracy of 86.3%, AUC of 92.4%, and F1-score of 84.7%.

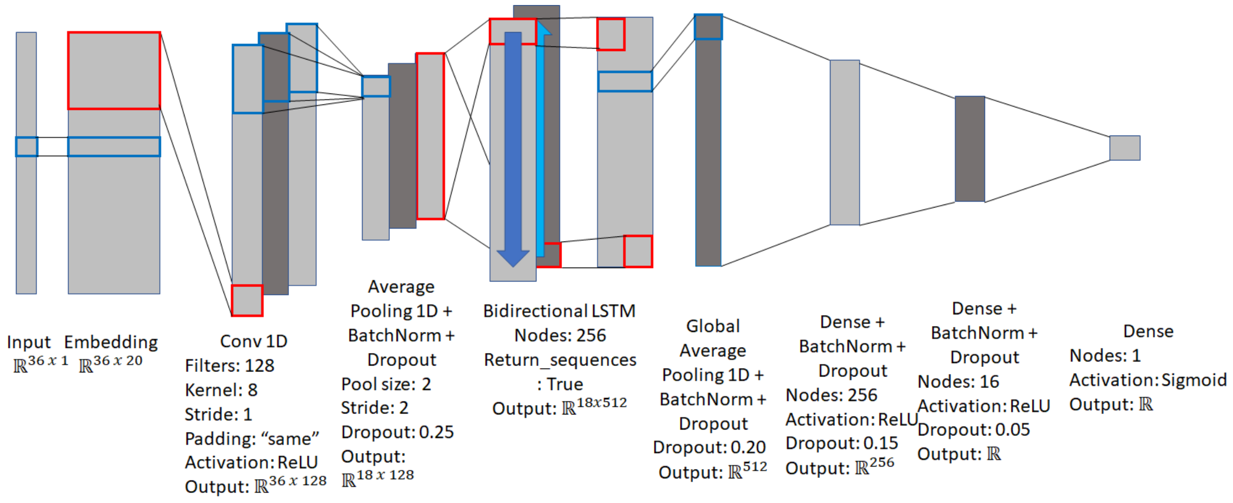

Fig B. Architecture of the second and third antifungal peptide classifiers based on the classifier described in Sharma et al. *[7]*, **using a CNN-biLSTM architecture.**

Hemolytic Peptide Classifier

The hemolytic peptide classifier is based on the work by Yaseen et al. [10], but with a different network architecture. We used the same architecture as the model from the antifungal peptide classifier for the DCNN-biLSTM, using the input based on BLOSUM weights [9]. We were able to achieve comparable results to those referenced in the paper using this architecture. The model was trained using a five-fold random split (80% of the hemolytic peptide dataset was used for training and 20% was used for testing), as was done for Yaseen et al. [10]. The model was trained for 20 epochs with a batch size of 16 using the Adam optimizer with a learning rate of 0.03. The mean model accuracy was 77.9%, AUC was 85.9%, and F1-score was 78.3%, as compared to the 88% AUC reported in Yaseen et al. [10]. We used the model trained on the first fold, which was the best

performing model on two of the three metrics: accuracy of 81.1%, AUC of 89.1%, and F1-score of 78.3%.

Fung-AI Model

As described in the main text, our generative adversarial network (GAN) is a generative model framework composed of two neural networks, a generator *G,* and a discriminator, *D*. Unlike standard GANs, our model, shown in **Fig C**, has an additional encoder that pairs with the generator, which in turn acts as both a generator and a decoder, as it serves the same purpose for both a GAN and an autoencoder, to take in some input q or z and output a real looking antifungal peptide sequence. As the dataset was somewhat small, and resulted in poor performance when trained solely on antifungal peptides, we did two things to create a larger dataset for training the GAN. First, we trained on both anti-fungal and non-antifungal peptides, accounting for this in the discriminator loss function. Second, we trained an autoencoder while simultaneously training the generator, which had the benefit of reducing mode collapse. The model was forced to still be able to reconstruct real antifungal and non-antifungal peptides, thereby enforcing meaning in the embedding space. As a result, there are three models that compose the GAN: the discriminator, the generator/decoder, and the encoder.

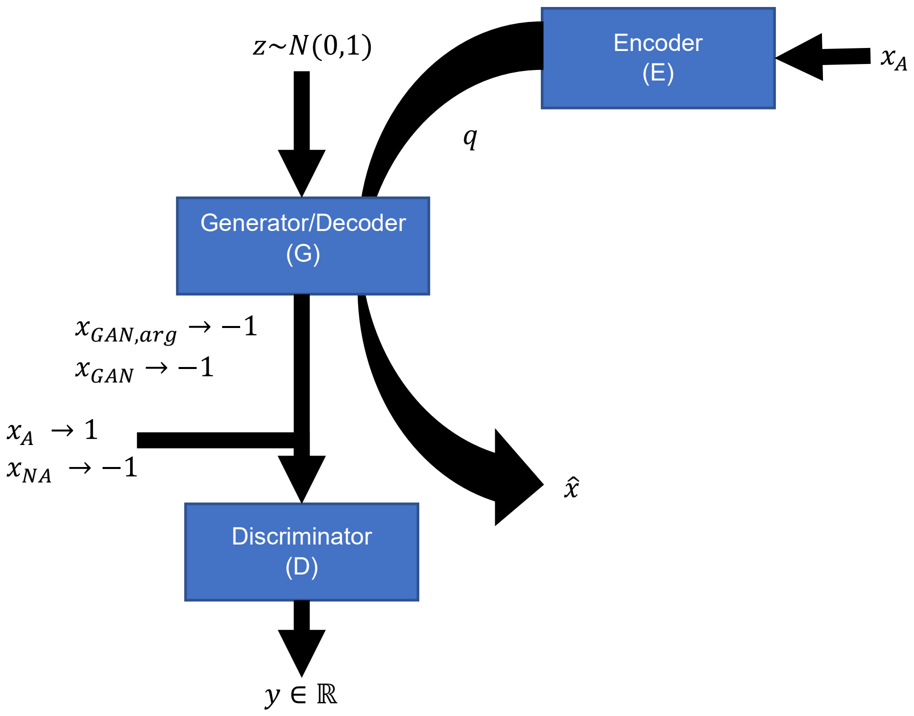

Fig C. Architecture of the GAN and encoder that is used for generating novel antifungal peptides. The GAN is composed of the discriminator, D, and the generator/decoder, G. The autoencoder is composed of the encoder, E, and the generator/decoder. When training the discriminator, the real non-antifungal peptides (x_{NA}), and the softmax output (x_{GAN}) and the argmax output ($x_{GAN,\ arg}$) sequences generated by the generator/decoder had a target value, y, of -1, while real antifungal peptides (x_A) had a target value, y, of 1. The encoder takes input x_A, which is a peptide sequence, encodes it as a vector q and outputs a reconstructed version of that peptide $\hat{x}$ with the goal that $x = \hat{x}$. Similarly, the

generator/decoder, G, takes in a vector of normal distributed noise, $z \sim N(0,1)$, with the same dimensions as q, and creates a new peptide.

The Encoder

The maximum number of words included in the embedding layer of the encoder, E, was 25, which was selected by the number of peptides plus additional start, stop, unknown, and empty tokens. The input to the model was a tokenized version of the peptide where each token is an amino acid. Additionally, we selected an embedding size of 200. The encoder portion was a CNN with three convolutional layers with 128, 256, and 512 filters, all of which used a ReLU activation function, a kernels of 5, and strides of 2 (**Fig D**). Between the convolutional layers were batch normalization layers. The final layer of the encoder was a global pooling layer. As a result, the encoding space of any peptide is a 512-dimensional vector.

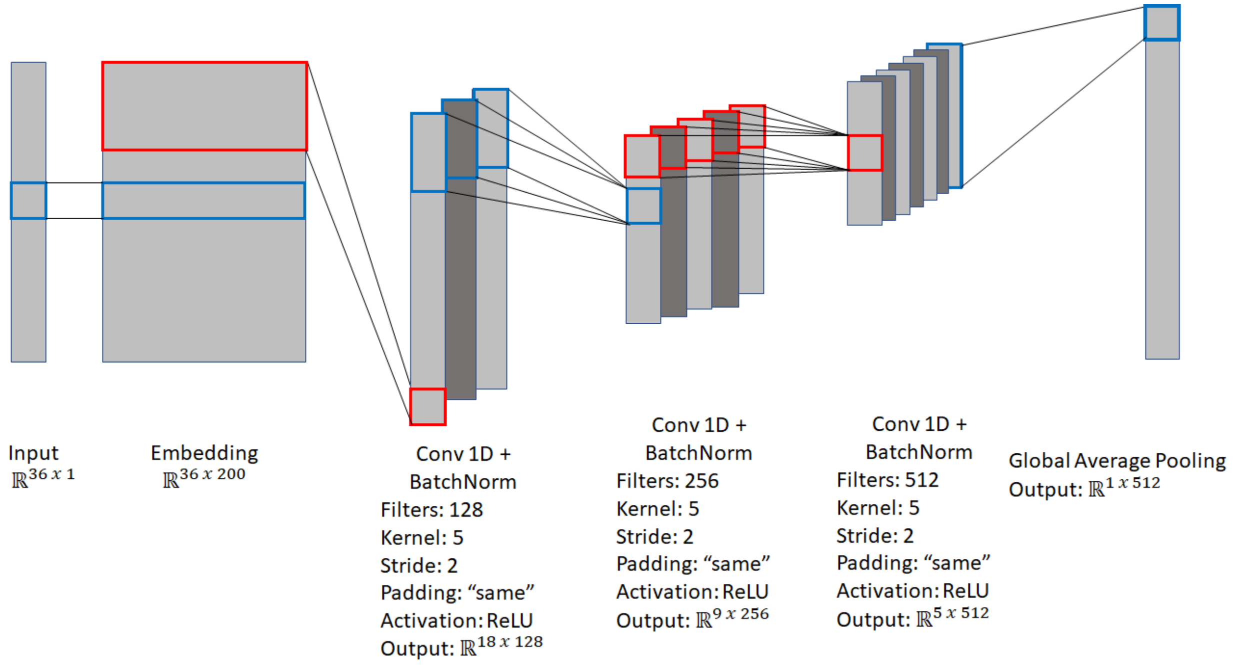

Fig D. Architecture of the encoder of the Fung-AI GAN.

The Generator/Decoder

As shown in **Fig E**, the generator/decoder, G, consists of alternating convolutional layers and up-sampling layers. The input is a 512-dimensional vector that should be normally distributed. The convolutional layers had filter numbers of 512, 256, 128, and 32 and all used a ReLU activation function and a kernel of 3. The up-sampling layers consisted of a kernel of 3, 3, 2, and 2. These layers were followed by a final convolutional layer with filter number of 32, a ReLU activation function, and a kernel of 2. The final layer consisted of a dense fully connected layer with an output size of 23 using a softmax activation function.

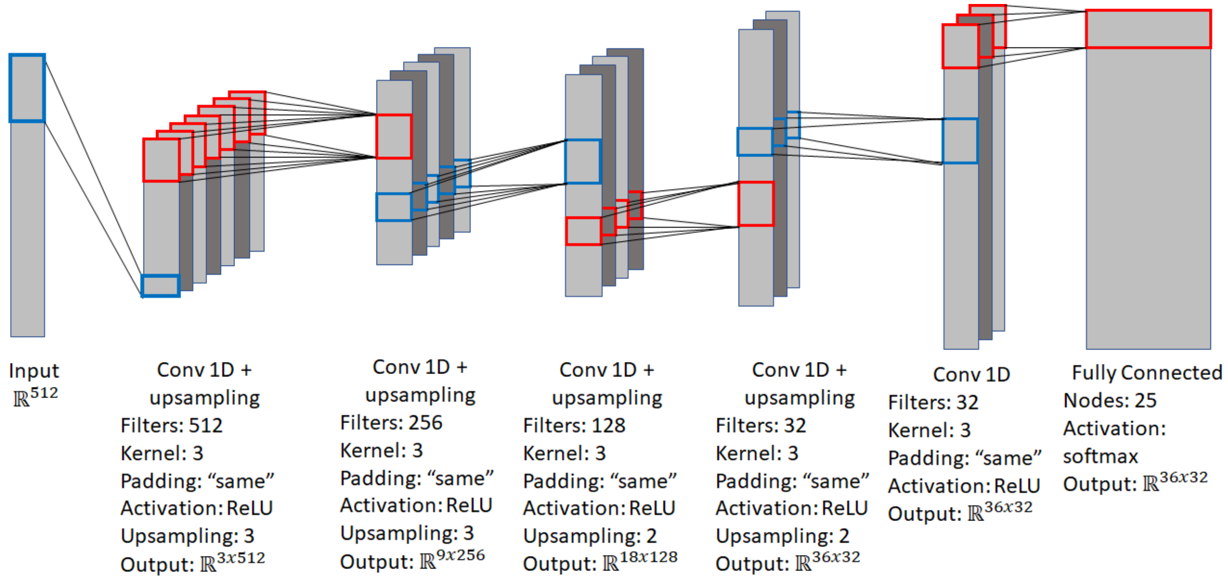

Fig E. Architecture of the decoder/generator of the Fung-AI GAN

The Discriminator

The structure of the discriminator, D, is different from the encoder paired with the generator in two ways. The discriminator uses a bidirectional long short-term memory (LSTM) rather than several convolutional blocks. We opted for this architecture since the antifungal peptide classification tasks using a bidirectional LSTM achieved good performance. The substitution also reduced the complexity of the GAN substantially. Additionally, the encoder uses a standard embedding layer while the discriminator uses a dense layer without a bias. This change was made because the output of the generator at each position of the peptide sequence is a probability vector with dimension $\mathbb{R}^{d \times 1}$. This probability vector cannot be used as input to an embedding layer without losing differentiability of the loss function through using an argmax function, thus preventing the backpropagation necessary to train the generator. Therefore, the embedding layer is swapped out for a dense layer with a linear activation function and no bias term that takes as input a vector with dimensions $\mathbb{R}^{d \times 1}$. The result is a layer that is analogous to an embedding layer that produces a linear combination of the embeddings and allows for backpropagation.

The structure of the discriminator was the dense fully connected layer without bias, as described above, with an output size of 200, sharing the embedding weights of the pretrained autoencoder as a starting point. This layer fed into a bidirectional LSTM with a hidden dimension of 128, resulting in a vector of 512 dimensions for each peptide. This vector then fed into a series of three blocks of dropout, batch normalization, and dense fully connected layers. All three blocks used a dropout of 0.2. The first block used a dense layer of 128 with leaky ReLU(0.1). The second block used a dense layer of 64 with a leaky ReLU(0.1). The final layer is a dense linear layer with an output dimension of 1. The structure of the discriminator is provided in **Fig F**.

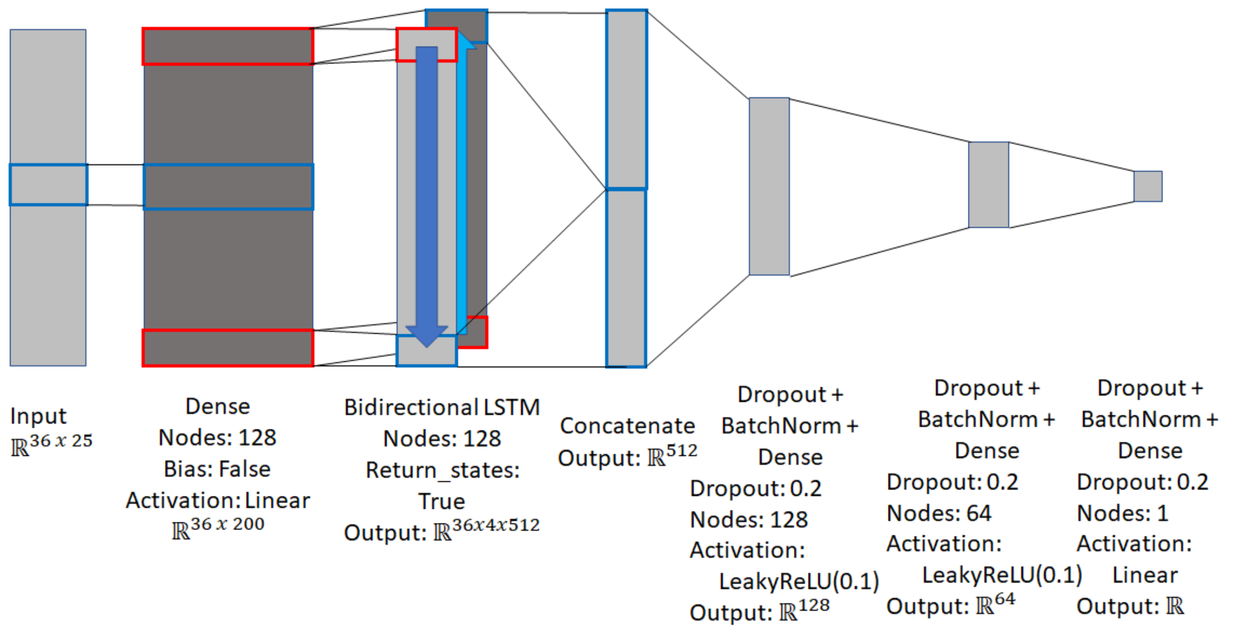

Fig F. Architecture of the discriminator in the Fung-AI GAN.

Fung-AI Loss Function

The Generator

While training the generator, G, using random noise passed through the entire GAN, the autoencoder composed of the encoder-decoder was being trained simultaneously with the generator acting as a decoder. Therefore, there are two inputs to the model, $z \sim N(\mathbf{0}, \mathbf{1})$ and x which is a real peptide sequence, either antifungal or not-antifungal. As a result, the loss function for training the generator was as follows:

$$\mathcal{L}_G = \mathcal{L}_{CE} + \mathcal{L}_{KL} + \mathcal{L}_W \tag{3}$$

The first two terms of the loss function correspond to the training of the autoencoder where $\mathcal{L}_{CE}$ is the cross entropy of the reconstructed sequence x, $\mathcal{L}_{KL}$ is the Kullback-Leibler divergence of the embedding of x with a standard normal distribution, and $\mathcal{L}_W$ is the Wasserstein loss for training the GAN.

The loss term $\mathcal{L}_{CE}$ in equation 3 is the cross-entropy loss of the encoder-decoder reconstruction and is defined as:

$$\mathcal{L}_{CE} = -\frac{1}{N}\sum_{i=0}^{N}\sum_{x \in X_i}^{T_i} p(x) log\big(q(x)\big)$$

and is applied to the reconstructed sequence outputs, shown in **Fig C** as $\hat{x}$, where $\hat{x} = G(E(x))$. The $\mathcal{L}_{CE}$ loss term ensures the encoder-decoder is capable of maintaining a meaningful embedding space. We found that without this term, the model experienced mode collapse fairly quickly.

The loss term $\mathcal{L}_{KL}$ function in equation 3 is the Kullback-Leibler divergence applied to embedding representation of the embedding vector $\hat{z}$ of sequence x, where $q = E(x)$. While the cross-entropy loss term helps to constrain the model to have meaning, this term ensures that this space matches with the standard normal noise input into the GAN. The generalized form of the Kullback-Leibler divergence is:

$$\mathcal{L}_{KL}(P||Q) = \sum_{x \in X} P(x) \log\left(\frac{P(x)}{Q(x)}\right)$$

However, as we wish to constrain the distribution of the embedding vectors q of the known antifungal sequences x such that they have a standard normal distribution and match the input noise to the GAN z, the loss function simplifies to

$$\mathcal{L}_{KL}(P||Q) = \frac{1}{N}\sum\left[\log\left(\frac{\sigma_P}{\sigma_Q}\right) + \frac{\sigma_P^2 + \left(\mu_P - \mu_Q\right)^2}{2\sigma_Q^2} - \frac{1}{2}\right] = \frac{1}{N}\sum_{q} \log\left(\frac{1}{\sigma_Q}\right) + \frac{1 + \mu_Q^2}{2\sigma_Q^2} - \frac{1}{2}$$

since $\sigma_P = 1$ and $\mu_P = 0$ [5]. Therefore, where $\sigma_E = std(q) = std(E(x))$ and $\mu_E = \frac{1}{N}\sum_i^N q_i = \frac{1}{N}\sum_i^N E(x)_i$,

$$\mathcal{L}_{KL} = \frac{1}{N}\sum_{N} \log\left(\frac{1}{\sigma_E}\right) + \frac{1 + \mu_E^2}{2\sigma_E^2} - \frac{1}{2}$$

In addition, in accordance with previous GAN work, we use a Wasserstein loss function rather than a binary cross entropy loss function

$$\mathcal{L}_W = \frac{1}{N}\sum_{n=1}^{N} y_n * \hat{y}_n.$$

where y_n is the ground truth value of the inputs sequence, $y_n = \{-1,1\}$, and $\hat{y}_n$is the predicted value from the output of the discriminator through a linear activation function [11] . When training the generator, the target value of $y_n = 1$.

Finally, after each epoch, the loss weights are adjusted so as to ensure the Wasserstein loss does not overtake the other two components. To do this, we set the ratio of the three losses $(\mathcal{L}_W, \mathcal{L}_{CE}, \mathcal{L}_{KL}) = \left(1, \left|\frac{\mathcal{L}_W}{L_{CE}}\right|, \left|\frac{\mathcal{L}_W}{L_{CE}}\right|\right)$. We found that this resulted in slower, but far more stable training. The loss weighting is initialized to (1,1,1).

The Discriminator

The loss function for the discriminator has one component:

$$\mathcal{L}_D = \mathcal{L}_W$$

the Wasserstein Loss, $\mathcal{L}_W$, as described above for the generator. The discriminator was trained using the approach took in training a text-to-image GAN [12]. The authors trained the model on generated data ($y = -1$) and real data ($y = 1$), but also on data in which real images were randomly

assigned to a non-relevant text pair ($y = -1$) so the GAN would not generate reasonable images that did not match the text. We adapted this idea and trained the discriminator on non-antifungal peptides ($y = -1$) to provide real negative examples for the GAN to be able to discriminate between sequences we are interested in and sequences we are not interested in.

Additionally, the discriminator was trained on two types of generated data, both sequences that had and did not have an argmax function applied to each peptide location. As a result, generated data input into the discriminator could appear as a discrete peptide at each location, or a distribution. The addition of training on the softmax distribution output of the generator allowed for more controlled and stable training as the distribution form more closely mirrored the input to the discriminator when training the generator. Therefore, the model was trained on 3 batches of negative data and two batches of positive data.

Peptide Generation

In the experiment, 10,000 peptides were generated using a standard normal distribution for noise generation as input to the generator. In order to increase diversity of the generated sequences, rather than using an argmax function exclusively to sample the output of the softmax function from the generator, we opted for a stochastic approach. In this approach, the token with the highest probability $\arg\max(\hat{\boldsymbol{y}}_{\boldsymbol{i}})$ is chosen (1-m)% of the time. The rest of the m% of the time, the token is selected by drawing from a multinomial distribution is sampled, where $\hat{\boldsymbol{y}}_{\boldsymbol{i}}$ acts as the probability vector parameter. As this is a variation on the sequence made by random chance, we call this parameter m the mutation rate. Determining which tokens are selected using the argmax and which are selected using the multinomial distribution is done by generating a random number between 0 and 1, drawn from a uniform distribution, for each position in the sequence. When the random number at position i is greater than the mutation rate, or the highest probability token is a start or end token, the argmax is used. When it is below the mutation rate, the multinomial sampling is used. We used a mutation rate of $m = 0.1$. As a result, there were zero repeated peptides in the 10,000, and there was no overlap in the training or test data with the generated peptides. All peptides were within the desired range of 10-35 amino acids in length. A total of six peptides were discarded from the 10,000 for not ending on an end token.

Supplemental References:

[1] M. Abadi *et al.*, "TensorFlow: Large-Scale Machine Learning on Heterogeneous Distributed Systems," Mar. 16, 2016, *arXiv*: arXiv:1603.04467. doi: 10.48550/arXiv.1603.04467.

[2] F. Chollet and others, "Keras." 2015. [Online]. Available: https://keras.io

[3] P. Remy, "Temporal Convolutional Networks for Keras," *GitHub repository*. GitHub, 2020. [Online]. Available: https://github.com/philipperemy/keras-tcn

[4] D. P. Kingma and J. Ba, "Adam: A Method for Stochastic Optimization," Jan. 30, 2017, *arXiv*: arXiv:1412.6980. doi: 10.48550/arXiv.1412.6980.

[5] D. I. Belov and R. D. Armstrong, "Distributions of the Kullback-Leibler divergence with applications," *Br. J. Math. Stat. Psychol.*, vol. 64, no. Pt 2, pp. 291–309, May 2011, doi: 10.1348/000711010X522227.

[6] V. Singh, S. Shrivastava, S. Kumar Singh, A. Kumar, and S. Saxena, "Accelerating the discovery of antifungal peptides using deep temporal convolutional networks," *Brief. Bioinform.*, vol. 23, no. 2, p. bbac008, Mar. 2022, doi: 10.1093/bib/bbac008.

[7] R. Sharma, S. Shrivastava, S. Kumar Singh, A. Kumar, S. Saxena, and R. Kumar Singh, "Deep-AFPpred: identifying novel antifungal peptides using pretrained embeddings from seq2vec with 1DCNN-BiLSTM," *Brief. Bioinform.*, vol. 23, no. 1, p. bbab422, Jan. 2022, doi: 10.1093/bib/bbab422.

[8] S. Bai, J. Z. Kolter, and V. Koltun, "An Empirical Evaluation of Generic Convolutional and Recurrent Networks for Sequence Modeling," Apr. 19, 2018, *arXiv*: arXiv:1803.01271. doi: 10.48550/arXiv.1803.01271.

[9] H. ElAbd, Y. Bromberg, A. Hoarfrost, T. Lenz, A. Franke, and M. Wendorff, "Amino acid encoding for deep learning applications," *BMC Bioinformatics*, vol. 21, no. 1, p. 235, June 2020, doi: 10.1186/s12859-020-03546-x.

[10] A. Yaseen, S. Gull, N. Akhtar, I. Amin, and F. Minhas, "HemoNet: Predicting hemolytic activity of peptides with integrated feature learning," *J. Bioinform. Comput. Biol.*, vol. 19, no. 5, p. 2150021, Oct. 2021, doi: 10.1142/S0219720021500219.

[11] M. Arjovsky, S. Chintala, and L. Bottou, "Wasserstein gan," *ArXiv Prepr. ArXiv170107875*, 2017.

[12] S. Reed, Z. Akata, X. Yan, L. Logeswaran, B. Schiele, and H. Lee, "Generative Adversarial Text to Image Synthesis," June 05, 2016, *arXiv*: arXiv:1605.05396. doi: 10.48550/arXiv.1605.05396.
